# Federated Learning for Human Pose Estimation on Non-IID Data via Gradient Coordination

**DOI:** 10.3390/s25144372

**Published:** 2025-07-12

**Authors:** Peng Ni, Dan Xiang, Dawei Jiang, Jianwei Sun, Jingxiang Cui

**Affiliations:** 1 School of Applied Technology, Changchun University of Technology, Changchun 130012, China; nipeng@ccut.edu.cn (P.N.); jiangdawei@ccut.edu.cn (D.J.); 2 College of Computer Science and Engineering, Changchun University of Technology, Changchun 130012, China; 2202403135@stu.ccut.edu.cn; 3 School of Mechatronic Engineering, Changchun University of Technology, Changchun 130012, China; 4 School of Mathematics, Jilin University, Changchun 130015, China; cuijx1021@mails.jlu.edu.cn

**Keywords:** human pose estimation, federated learning, gradient coordination, non-IID data

## Abstract

Human pose estimation is an important downstream task in computer vision, with significant applications in action recognition and virtual reality. However, data collected in a decentralized manner often exhibit non-independent and identically distributed (non-IID) characteristics, and traditional federated learning aggregation strategies can lead to gradient conflicts that impair model convergence and accuracy. To address this, we propose the Federated Gradient Harmonization aggregation strategy (FedGH), which coordinates update directions by measuring client gradient discrepancies and integrating gradient-projection correction with a parameter-reconstruction mechanism. Experiments conducted on a self-constructed single-arm robotic dataset and the public Max Planck Institute for Informatics (MPII Human Pose Dataset) dataset demonstrate that FedGH achieves average Percentage of Correct Keypoints (PCK) of 47.14% and 66.31% across all keypoints, representing improvements of 1.82 and 0.36 percentage points over the Federated Adaptive Weighting (FedAW) method. On our self-constructed dataset, FedGH attains a PCK of 86.4% for shoulder detection, surpassing other traditional federated learning methods by 20–30%. Moreover, on the self-constructed dataset, FedGH reaches over 98% accuracy in the keypoint heatmap regression model within the first 10 rounds and remains stable between 98% and 100% thereafter. This method effectively mitigates gradient conflicts in non-IID environments, providing a more robust optimization solution for distributed human pose estimation.

## 1. Introduction

Human pose estimation is an important downstream task in computer vision. It involves detecting joint keypoints from images or video frames and inferring the overall body pose. This capability underpins applications such as action recognition, virtual reality, and human–computer interaction. In medical robotic-assisted rehabilitation scenarios, pose estimation enables real-time monitoring of patient movements, thereby providing quantitative assessment and decision-support throughout the recovery process. The fundamental principle of pose estimation typically employs deep convolutional neural networks to regress or predict each joint’s two-dimensional or three-dimensional coordinates via heatmap representations.

In recent years, deep learning-based methods have achieved remarkable progress on public datasets such as MPII and Common Objects in Context (COCO) [1], with most advances relying on centrally aggregating all annotated data in the cloud for training. In real-world deployments, pose images and their keypoint annotations are collected by diverse cameras and wearable sensors. These data naturally remain distributed across multiple clients and tend to follow non-IID distributions. Moreover, centralizing raw images raises serious privacy and compliance concerns because they can reveal sensitive information such as individual behaviors and physiological traits. Moreover, the massive volume of video streams and high-resolution images imposes substantial bandwidth and communication costs. Under these circumstances, the inability to share data centrally across devices and sensors leads to limited model generalization in new environments and makes models susceptible to local overfitting. It can also cause gradient conflicts during training, resulting in a sharp decline in keypoint-detection accuracy.

In the field of computer vision, federated learning has demonstrated significant advances in image classification, object detection, and semantic segmentation, validating its capacity for model coordination and privacy protection in non-IID scenarios [2,3]. In human pose estimation, the Federated Learning for Human Pose Recognition (FL-HPR) multi-sensor fusion framework achieves high-precision keypoint detection on the non-IID MPII dataset [4].

Federated learning enables collaborative model training via iterative communication between a central server and numerous clients. Initially, the server broadcasts a global model. Each client then trains locally on its private data and sends model updates back. The server aggregates these updates to refine the global model, and this cycle continues until convergence. This workflow enables collaborative optimization of pose-estimation models without sharing raw images. For example, the Cooperative Camera federated Learning Platform (COALA) platform supports federated pose-estimation training and evaluation on MPII, COCO, and other benchmarks [5]. The Federated Computer Vision framework (FedCV) provides a unified federated learning benchmark that compares non-IID performance across classification, segmentation, detection, and pose-estimation tasks [6]. A recent survey comprehensively summarizes the applications and challenges of federated learning in human pose estimation, demonstrating its feasibility [7].

Federated learning applied to human pose estimation enables multi-device collaborative training under user-privacy constraints. However, pose images collected by different clients may vary in viewpoint, illumination, and action distribution. Such heterogeneity can cause local update gradients to conflict with one another, severely impairing global model convergence and keypoint-detection accuracy. Gradient conflict can be formalized by considering the angle between two clients’ gradient vectors. If this angle is obtuse, their aggregated update may cancel out or reverse the intended direction. As a result, model performance suffers, especially under non-IID data distributions.

Zhang et al. examined a multi-camera setup where client A mainly records side-view poses, whose right-shoulder gradient updates tend to converge inward. Client B, capturing frontal views, generates outward-pointing right-shoulder gradients. When using Federated Averaging (FedAvg) to aggregate these updates, the conflicting gradients can cancel each other out. This cancellation reduces update strength and substantially lowers Percentage of Correct Keypoints at a distance threshold of 0.5 (PCK@0.5) for shoulder and wrist keypoints [8]. Filippos et al. further show that gradient conflict leads to sluggish convergence and oscillating validation loss over aggregation rounds. They also report a 10–15% decrease in elbow and wrist localization accuracy for FedAvg-based distributed pose-estimation models [9]. Moreover, gradient conflict may lead to client-specific overfitting or underfitting, exacerbating overall performance instability.

To address the aforementioned challenges, researchers have proposed various federated aggregation strategies. FedAvg [10] uses simple weighted averaging of local updates. Federated Proximal (FedProx) [11] introduces a proximal term to constrain local model drift. Federated Batch Normalization (FedBN) [12] retains batch-norm statistics on the client side to accommodate distributional shifts. More recently, FedAW [13] dynamically adjusts server-side aggregation weights based on local dataset quality, and Federated Learning with Dynamic Regularization (FedDyn) [14] employs a correction term to counteract client drift under non-IID data by aligning local and global gradients. Each strategy aims to mitigate the adverse effects of data heterogeneity on convergence and model generalization.

However, human pose estimation introduces unique challenges that exacerbate gradient conflict during aggregation. First, the prediction of high-resolution heatmaps for each keypoint imposes strong spatial coherence constraints—clients with disparate pose distributions (e.g., athletic versus surveillance data) may generate heatmap gradients that pull peaks in incompatible directions. Second, pixel-wise regression losses (e.g., mean-squared error or Gaussian-based likelihood) heavily penalize even minor localization errors, so small directional disagreements in these fine-grained gradients can cancel each other out when averaged. Third, variability in subject scale and occlusion patterns demands specialized receptive-field adaptations, causing clients focused on large versus small figures to produce updates favoring different network branches. Finally, the anatomical dependencies among keypoints (for example, the kinematic relationship between elbow and wrist) introduce non-local correlations across heatmaps, meaning that independent client updates often tug the model in conflicting anatomical directions. Collectively, these structured, high-resolution outputs and strong spatial and anatomical couplings render simple averaging slow, unstable, or even detrimental.

To this end, this paper introduces an improved aggregation algorithm based on gradient coordination—FedGH—built upon the FedAW framework. This method first mitigates conflicting gradients by measuring the inner product discrepancies among client gradients and applying the Projected Conflicting Gradients (PCGrad) projection-correction mechanism. Then, during the parameter-reconstruction phase, it performs weighted reconstruction and aggregation by integrating the corrected gradient information with client sample size weights. This approach effectively reduces global gradient conflict while preserving the personalized updates of each client. In addition, FedGH is integrated into the training pipeline of the PoseResNet model based on ResNet50. By adopting asynchronous updates and dynamic client-selection strategies, the proposed method addresses system heterogeneity and communication delays.

Experimental results demonstrate that the proposed method achieves significant performance gains on both a self-constructed single-arm robotic human pose dataset and the MPII [15] benchmark. Our approach not only accelerates convergence but also improves keypoint-detection accuracy in terms of average PCK, consistently outperforming several baseline methods including FedAvg, FedProx, FedDyn, FedBN, and FedAW. Our experiments demonstrate that the gradient-coordinated FedGH aggregation strategy is both universal and robust across diverse heterogeneous datasets. The approach consistently enhances keypoint-detection performance and ensures stable convergence in federated human pose-estimation tasks.

The main contributions of this paper are as follows.

- We propose FedGH, a gradient-coordination aggregation strategy specifically designed for keypoint-regression tasks in human pose estimation, and demonstrate its effectiveness by integrating it into a federated learning framework. FedGH employs a gradient-discrepancy metric and the PCGrad projection-correction mechanism within the FedAW aggregation process, followed by parameter reconstruction via weighted averaging to resolve conflicting gradient directions and optimize global updates. We further integrate FedGH into the PoseResNet (ResNet50 backbone) training pipeline, augmented with asynchronous updates and dynamic client selection, to address network heterogeneity and communication delays—thereby enhancing overall system robustness and efficiency.
- We present a novel single-arm robotic rehabilitation pose dataset consisting of 2060 high-resolution images of a single subject, captured from four fixed viewpoints (anterior, posterior, left lateral, right lateral). The images cover both upright standing and arm-extended postures, with a single-arm manipulator placed over the limb—thereby covering the wrist, elbow and shoulder regions. Acquisition was performed under consistent lighting, background and camera settings to balance stability and scene variation. Three keypoints (shoulder, elbow, wrist) were annotated using a combined visual-marker and manual verification workflow to ensure high spatial accuracy. This dataset enables rigorous assessment of FedGH in practical robot-assisted rehabilitation contexts.
- We conduct extensive experiments on both the self-constructed single-arm robotic pose dataset and the MPII benchmark using the average PCK metric. We systematically compare FedAvg, FedBN, FedProx, FedDyn, FedAW, and FedGH to demonstrate the effectiveness and generalization of FedGH across heterogeneous scenarios.

The remainder of this paper is organized as follows: Section 2 reviews related work; Section 3 details the FedGH method; Section 4 presents experimental design and results; and Section 5 concludes and outlines future research directions.

## 2. Related Work

### 2.1. Federated Learning

Federated learning is a privacy-preserving distributed machine learning paradigm that achieves collaborative training in multi-source heterogeneous environments by exchanging model updates rather than raw data. Since McMahan et al. proposed the FedAvg algorithm [10], the field of federated learning has continued to evolve. Kairouz et al. present a comprehensive survey of federated learning algorithms, systems, and privacy challenges, thereby providing the research framework and evaluation criteria for this study [16].

FedAvg updates the global model under independent and identically distributed (IID) data distributions by weighted averaging of clients’ local model parameters, but in non-IID scenarios it is prone to conflicts in client update directions, leading to slower global convergence or even performance degradation. To alleviate this issue, Li et al. proposed FedProx, which adds a regularization term to the local objective to limit the deviation of local models from the global model, thereby mitigating drift caused by data heterogeneity [11]. Li et al. also proposed FedBN, which retains batch normalization statistics on the client and does not update local BN parameters during aggregation. This design effectively reduces performance degradation caused by differing feature distributions across clients [12]. More recently, Acar et al. introduced FedDyn, a federated learning method based on dynamic regularization that adaptively corrects local update directions to further counteract the adverse effects of non-IID data [14].

The FedAW algorithm takes a different approach. On the server side, it dynamically assigns aggregation weights based on each client’s data quality. This weighting ensures that updates from more reliable sources have a stronger influence on the global model. As a result, FedAW significantly accelerates convergence and boosts final performance under non-IID conditions [13].

To address gradient conflict, a variety of approaches have been proposed. Karimireddy et al.’s Stochastic Controlled Averaging for Federated Learning (SCAFFOLD) employs control variates to correct update bias between the server and clients, reducing the variance between local updates and the global direction and thereby accelerating convergence [17]. Yu et al. introduced PCGrad, which uses pairwise gradient inner-product projection to correct conflicts by projecting opposing gradients onto non-conflicting subspaces, significantly enhancing aggregation stability [18]. Additionally, clustering-based grouped aggregation strategies have been developed, partitioning clients with similar data distributions into the same group for local aggregation to mitigate heterogeneity effects [19].

Adaptive client-selection mechanisms dynamically determine each round’s participating client set based on update quality and availability, balancing communication overhead and model performance. While federated learning has made significant advances in model aggregation, gradient coordination, client selection, and communication acceleration, these studies have not considered integration with human pose estimation. Under the highly non-IID characteristics of pose-estimation tasks, reducing gradient conflict while ensuring aggregation efficiency and model generalization remains a pivotal research challenge.

### 2.2. Human Posture Estimation

Human pose estimation aims to precisely locate human joint keypoints from images or videos and infer the overall body pose. Image-based human pose detection has reached a considerable level of maturity. Early regression-based methods include DeepPose, proposed by Toshev and Szegedy, which employs a deep neural network to automatically learn effective feature representations via convolutional neural networks (CNNs) and directly regress joint positions [20]. By leveraging an end-to-end training framework, DeepPose improved the accuracy and robustness of keypoint localization, addressing the precision limitations and high complexity of traditional pose-estimation algorithms. Subsequently, Newell et al. introduced the heatmap-based Stacked Hourglass network, which achieves higher accuracy through multi-scale feature aggregation and iterative refinement [21]. Wei et al. proposed Convolutional Pose Machines, which progressively optimize spatial contextual information within a multi-stage convolutional architecture [22].

Although early methods such as DeepPose and Hourglass laid the groundwork for deep pose estimation, recent years have witnessed the emergence of models that balance real-time performance and accuracy. Cao et al. proposed High-Resolution Network for Multi-Person Pose Estimation (HRNet-MultiPose), which achieves high-precision detection for real-time multi-person pose estimation [23]; Xu et al. introduced Vision Transformer Pose Estimation (ViTPose), leveraging a vision-transformer architecture to further enhance robustness in complex environments [24].

In the high-resolution era, Wang et al. proposed HRNet, which maintains high-resolution representations by exchanging parallel high- and low-resolution feature maps, thereby improving keypoint localization accuracy [25]. Xiao et al. introduced a simple yet effective baseline, PoseResNet, for human pose estimation and tracking: built upon ResNet50, PoseResNet adds deconvolutional upsampling modules and employs a pre-trained convolutional backbone with simple fully connected regression layers, streamlining both model architecture and training. By using heatmap regression to generate high-resolution heatmaps and predicting joint locations from these maps, it better handles occlusions and complex poses, enhancing generalization and robustness and boosting pose-estimation accuracy [26]. Brasó et al. presented CenterGroup, an end-to-end attention-based multi-person pose-estimation method that reformulates joint grouping as a center-point-to-joint attention matching problem, overcoming the non-learnable clustering dependencies and inefficiencies of traditional bottom-up approaches and achieving higher accuracy and efficiency in crowded and occluded scenarios [27].

Although the aforementioned human pose-estimation methods perform exceptionally well on large-scale centralized datasets, centralized training poses practical challenges. Data-sharing requirements introduce privacy risks, and transmitting high-resolution images incurs substantial communication overhead. These issues are especially problematic in privacy-sensitive or bandwidth-limited settings. To this end, lightweight networks and efficient heatmap-decoding algorithms have been employed for on-device inference to accommodate resource-limited devices. In recent years, federated learning has been applied to human pose estimation to address these challenges. This paradigm enables collaborative training of keypoint detectors across multi-view cameras, wearable devices, and other distributed sensors. Crucially, raw images remain on local devices, preserving data privacy. For example, Qi et al. integrated a federated learning framework with human pose estimation for fall detection, demonstrating that multi-source data can enhance model generalization while preserving privacy [28].

Against this backdrop, the present study applies a federated gradient harmonization strategy to the human pose-estimation task based on PoseResNet. The FedGH method is designed to resolve gradient conflicts caused by pose diversity and background variations among clients. It harmonizes local gradients before aggregation, which accelerates convergence. Consequently, FedGH improves the accuracy of joint localization in federated human pose estimation.

## 3. Methodology

### 3.1. Framework Overview

**Figure 1:**
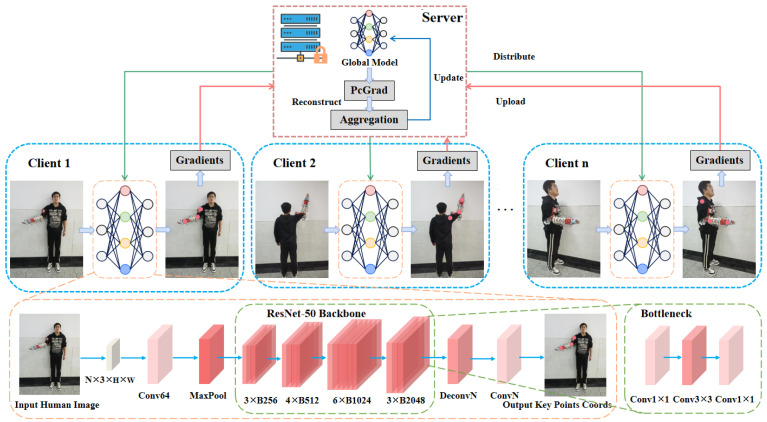
The proposed framework is based on Federated Learning (FL). In each communication round, clients download the global model from the server, perform local training, and compute gradients, which are then uploaded to the server. The server addresses gradient conflicts caused by data heterogeneity using the PCGrad projection, and updates the global model through parameter reconstruction and weighted aggregation.

In this study, a federated learning framework based on the FedGH method is constructed for the human pose-estimation task, as shown in Figure 1. In order to avoid gradient conflicts among multiple clients in non-IID scenarios, an improved federated learning aggregation method, FedGH, is adopted. Building upon the traditional FedAW framework, the FedGH method introduces a gradient difference metric and a projection-based correction mechanism. These enhancements aim to better coordinate the update directions of client models during global aggregation, thereby improving both the convergence efficiency and the generalization performance of the model in the human pose-estimation task.

Specifically, at the beginning of each training round (i.e., one communication cycle in which every client makes one pass over its entire local dataset and then synchronizes with the server), each client first downloads the latest global model from the server for initialization. After updating the model parameters on its local data, the client uploads the resulting gradient information back to the server. Subsequently, within the FedGH aggregation layer, the server computes the differences among the gradients of various clients and applies the PCGrad projection to correct conflicting gradients, alleviating inconsistencies in the update directions caused by data-distribution discrepancies. After the projection correction, FedGH further integrates the update results from all clients through a parameter-reconstruction technique and performs a weighted averaging to obtain a new global model.

Through this process, FedGH not only preserves the personalized update information of each client but also effectively reduces gradient conflicts at the global level, thereby achieving a more robust model performance. This study applies FedGH for federated learning in the human pose-estimation task and builds an optimized global model based on this method, providing a novel perspective and practical approach for distributed pose estimation.

### 3.2. Non-IID Data Partitioning

To simulate realistic discrepancies in data volume and distribution among clients, in this study we perform a non-IID partitioning of the entire sample set on the server according to client-specific weights. In our experiment, we assume K = 10 clients, and we preset a weight vector w = [0.30, 0.20, 0.15, 0.10, 0.08, 0.06, 0.04, 0.03, 0.02, 0.02] to reflect the proportion of data allocated to each client. In the partitioning function, the total number of samples is denoted by *n*, the minimum sample threshold is set to $m_{\min}=10$, and the random seed is fixed at 1234 to ensure reproducibility. The detailed procedure for non-IID data partitioning is as follows:

Prior to partitioning, we first normalize the preset client weight vector w to compute each client’s relative allocation ratio ${\tilde{w}}_{k}$, ensuring that the sum of all client allocation ratios equals 1, so that samples can subsequently be assigned proportionally:

(1) $${\tilde{w}}_{k}=\frac{w_{k}}{\sum_{i=1}^{K}w_{i}},\;k=1,\ldots ,K$$

Next, we allocate an initial number of samples $c_{k}$ proportionally; if any $c_{k}<m_{\min}$, we set $c_{k}\leftarrow m_{\min}$ to prevent insufficient samples from causing training failures:

(2) $$c_{k}=\left\lfloor {\tilde{w}}_{k}\;n\right\rfloor ,\;k=1,\ldots ,K$$

If $\sum_{k}c_{k}>n$, we iteratively reduce the surplus by one sample from each client in order (starting from client 1) until $\sum_{k}c_{k}=n$; if $\sum_{k}c_{k}<n$, we iteratively add one sample back to the earliest clients until the total matches *n*.

Finally, using the fixed random seed, we apply a pseudorandom shuffle to the full index list, and then slice and assign indices to each client according to the adjusted sample counts $c_{k}$:

(3) $${\mathrm{client\_indices}}_{k}=\mathrm{shuffled}\;[\;\sum_{i<k}c_{i}:\sum_{i\leq k}c_{i}\;]$$

This partitioning strategy not only strictly adheres to the preset client weights but also, through the minimum-sample constraint and total-volume adjustment, ensures unbiased preservation of the global sample count. As a result, it effectively simulates heterogeneous data volumes and distributions across clients, thereby providing a solid foundation for evaluating the robustness and optimization performance of FedGH under non-IID conditions.

### 3.3. FedGH Aggregation Strategy

Based on the FedAW aggregation algorithm and gradient-coordination algorithm, this paper employs a federated gradient-coordination strategy to address the issue of gradient conflicts during the aggregation process in federated learning. In a federated learning environment, due to the non-IID distribution of data across clients, the gradient directions computed by different clients may exhibit significant deviations, which can slow down the convergence of the global model or even degrade its performance. To mitigate this problem, the FedGH method is adopted; it introduces a gradient-projection-correction mechanism into the conventional FedAW framework to ameliorate gradient conflicts and enhance the optimization effect of the model.

#### 3.3.1. Gradient Computation

At the beginning of each communication round *t*, the server distributes the latest global model parameters $w_{t}$ to the clients. Each client *k* then trains the model on its local data and computes the gradient of the model parameters. The gradient computed by client *k* is defined as:

(4) $$g_{k}=\frac{w_{k}-w_{t}}{\eta }$$

where $w_{k}$ represents the model parameters of client *k* after local training, and η denotes the learning rate. Upon completion of the computation, the gradient information is uploaded to the server.

#### 3.3.2. Gradient-Projection Correction

In the federated learning setting, suppose that *K* clients participate in training in parallel, and the server obtains the set of client gradients $\left\{g_{1},g_{2},\ldots ,g_{K}\right\}$. Due to non-IID data distributions across different clients, the gradients computed by different clients may conflict with one another, resulting in a degradation of the global model update. To address this, FedGH applies pairwise projections to correct each client gradient $g_{k}$ during each round of aggregation, thereby mitigating gradient conflicts.

For each client *k*’s gradient $g_{k}$, the server randomly selects another client *j*’s gradient $g_{j}$ from the remaining K−1 gradients. If the inner product $\langle g_{k},\;g_{j}\rangle$ is negative, the gradients are oppositely directed, the gradient is corrected according to the PCGrad method as follows:

(5) $$g_{k}^{\prime }=g_{k}-\frac{\langle g_{k},g_{j}\rangle }{∥g_{j}{∥}^{2}}g_{j}\;\mathrm{if}\;\langle g_{k},g_{j}\rangle <0$$

FedGH employs entirely random sampling of gradient pairs instead of greedy selection or threshold-based filtering. This simple strategy not only reduces the complexity of hyperparameter tuning but also demonstrates superior robustness in experiments. Moreover, random sampling avoids biases introduced by fixed pairing patterns and prevents overcorrection of the most conflicting pairs, which could otherwise lead to information loss. This random pairing strategy also reduces the computational overhead from $O(K^{2})$ to approximately O(K), ensuring scalability of the aggregation stage as the number of clients grows substantially. Furthermore, in a multithreaded or distributed server environment, all clients projections can be executed in parallel without relying on a centralized global traversal, thereby achieving both high throughput and low latency.

Our ablation studies indicate that, in non-IID scenarios, the majority of global gradient conflicts stem from a small number of highly negative inner-product pairs; correcting these principal conflicts suffices to resolve the main discrepancies. Crucially, restricting each gradient to a single projection maximally preserves each client’s original update direction, avoiding the over-smoothing or attenuation of useful signals that would result from multiple projections. Thus, the pairwise projection correction achieves a balance between client-specific updates and global consistency, yielding simultaneous improvements in convergence speed and generalization performance under non-IID conditions.

#### 3.3.3. Parameter Reconstruction and Global Aggregation

After the gradient-projection correction, FedGH further employs a parameter-reconstruction method to fuse the updated results from all clients, followed by a weighted average to obtain the new global model. Specifically, the server performs a weighted summation of all corrected client gradients:

(6) $$g_{\mathrm{global}}=\sum_{k=1}^{K}\alpha_{k}g_{k}^{\prime }$$

where $\alpha_{k}$ represents the weight of client *k*, which is typically proportional to the size of its local dataset.

The final server updates the global model parameters:

(7) $$w_{t+1}=w_{t}+\eta g_{\mathrm{global}}$$

In order to more intuitively describe the inconsistency between client gradients, we introduce a gradient difference metric, defined as

(8) $$\Delta_{ij}=g_{i}-g_{j}$$

which quantifies the degree of difference between the gradients of any two clients.

Overall, the objective of the FedGH strategy is to minimize gradient conflicts among clients so that the global update can better integrate the information from all clients, thereby achieving improvements in both convergence speed and generalization performance. The optimization objective can be expressed as

(9) $$\min\sum_{i,j}I\left(g_{i}\cdot g_{j}<0\right)\left|g_{i}\cdot g_{j}\right|$$

where I(·) is an indicator function that takes the value of 1 when $g_{i}\cdot g_{j}$ is negative and 0 otherwise.

In summary, the FedGH aggregation strategy, by incorporating gradient difference measurement, PCGrad projection correction, and parameter-reconstruction techniques, effectively mitigates the conflict issues arising from inconsistent gradient directions among multiple clients in non-IID scenarios. This ensures that the global model converges more robustly in human pose-estimation tasks while enhancing overall generalization performance. This method not only preserves the individualized updates of each client but also achieves efficient global information fusion, providing a novel technical pathway and theoretical support for distributed pose estimation.

In this paper, to address the gradient conflicts caused by non-IID data distributions in federated learning, we adopt the FedGH aggregation strategy as an enhancement to the conventional FedAW algorithm.

Algorithm 1 delineates the aggregation procedure of FedGH, which is designed to mitigate inter-client gradient conflicts under non-IID conditions via gradient-coordination and parameter-reconstruction mechanisms. First, the server receives each client’s locally updated model parameters and, using the learning rate, converts them into gradient differences, forming the set *G*. To break any inherent ordering biases and to prevent overreliance on a fixed conflict-correction pattern, the algorithm randomly shuffles *G* at the start of each aggregation round, ensuring diversity and robustness in subsequent projection corrections. Next, for each gradient $g_{i}$ in the shuffled set, the algorithm computes its inner product with every other gradient $g_{j}$ in *G*. Whenever $\langle g_{i},g_{j}\rangle <0$, indicating opposing update directions, PCGrad is applied to project $g_{i}$ onto the orthogonal complement of $g_{j}$, thereby removing the conflicting component. This pairwise projection both rectifies significant negative conflicts and limits each gradient to a single correction step to avoid signal dilution from repeated adjustments. After all gradients have been corrected, the algorithm restores each client’s update delta from its modified gradient $g_{i}$ and reconstructs the corresponding client parameters. Subsequently, nonnegative weights are computed by measuring the inner products between each client’s update delta and the global “ideal” direction; if the sum of these weights is positive, they are normalized, otherwise the algorithm defaults to an equal-weight average. Finally, the server generates the new global model by computing a weighted sum of all client models according to these normalized weights.
 **Algorithm 1:** FedGH aggregation algorithm.  
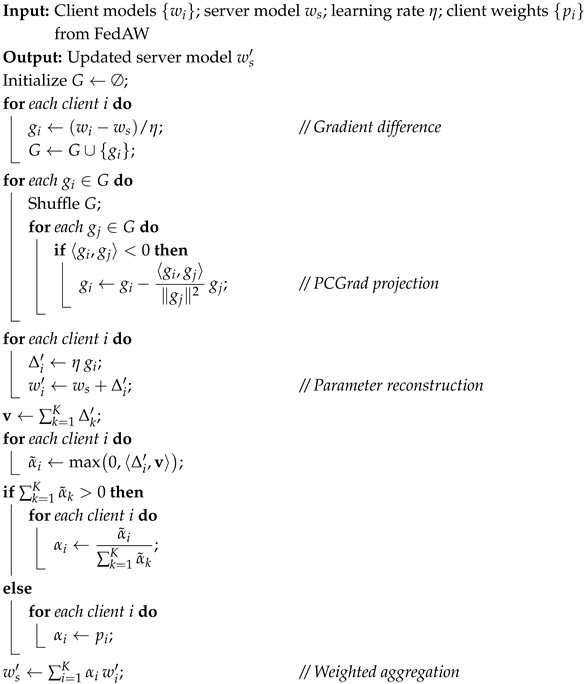


By combining random shuffling, pairwise projection, and dynamic weighting, FedGH effectively integrates information from heterogeneous clients—preserving personalized update advantages while substantially alleviating gradient conflicts in non-IID environments—thereby accelerating convergence and enhancing generalization. Moreover, FedGH is highly flexible in implementation, being agnostic to network architecture and data modality, which allows seamless integration into existing federated learning systems. Its generality makes it suitable for a wide range of federated learning applications, providing a more robust and efficient solution for distributed model training.

### 3.4. Federated Learning Framework for Human Pose Estimation

The federated learning framework proposed in this paper is designed to meet the dual requirements of large-scale, cross-device data fusion and privacy protection in human pose-estimation tasks. The framework ensures data security for all participating parties and enables collaborative training among multiple clients, thereby fully leveraging the diverse data resources collected from various locations to enhance the model’s ability to recognize pose features in complex scenarios. Each client conducts independent training on its local data to extract preliminary human pose features and subsequently exchanges model parameters with the server for knowledge sharing, without transmitting raw data. This effectively mitigates issues related to data silos and privacy breaches.

In the design of the framework, careful consideration is given to challenges that may arise in real-world deployments, such as network latency, device heterogeneity, and unstable communications. To address these challenges, asynchronous update mechanisms and dynamic client-selection strategies are adopted, ensuring that the global model can be updated iteratively in a stable and efficient manner in distributed environments. Moreover, the framework supports adaptive configuration of local training parameters, allowing each client to optimize its settings based on its hardware capabilities and data volume, thereby enhancing overall training efficiency.

Additionally, the framework exhibits strong modularity and scalability, which facilitates the seamless integration of various data preprocessing, feature extraction, and model optimization methods. This modular design lays a solid foundation for future deployment in a broader range of application scenarios.

## 4. Experiment

### 4.1. Dataset

**Figure 2:**
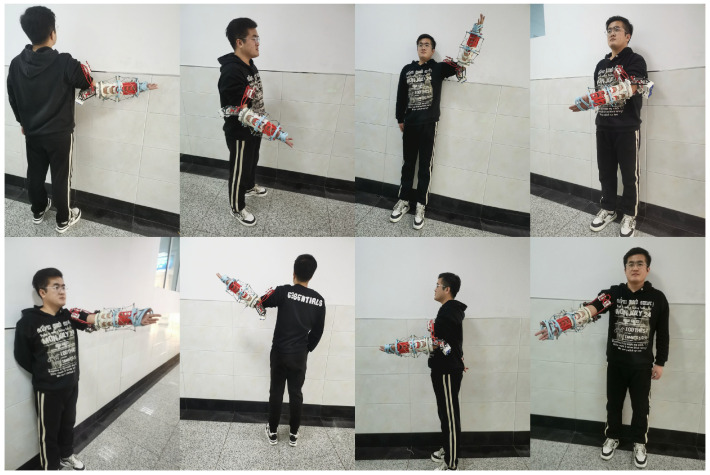
Example images from the self-constructed dataset.

In this study, we employ a self-constructed single-arm manipulator human standing-pose dataset alongside the public MPII human pose dataset as key benchmarks for experimental evaluation. The self-constructed dataset comprises 2060 images partitioned into 1427 for training, 409 for validation, and 224 for testing. Each sample illustrates a subject in an upright stance, with a single upper limb instrumented by a robotic manipulator (either left or right) occluding the wrist, elbow, and shoulder articulations, and is acquired from multiple camera perspectives (anterior, posterior, left lateral, and right lateral). Figure 2 presents several exemplar images from this dataset. During data acquisition, we rigorously controlled experimental lighting, background, and camera angles to ensure both stability and diversity of the collected data, thereby more faithfully reflecting the complexity and challenges of pose estimation in real-world application scenarios.

In terms of data annotation, we adopted a method combining visual markers with manual verification to accurately locate key body parts. Specifically, we focused on annotating three key points: the shoulder, elbow, and wrist. These three parts play a pivotal role in human motion analysis and robotic arm control. Through precise annotation of these three key points, we can not only more accurately capture the motion trajectory of the human upper limbs but also provide high-quality supervisory signals for subsequent algorithm training and testing. In the data preprocessing stage, the acquired pose images were normalized and rescaled to ensure uniformity, thus further enhancing the dataset’s practicality and generalizability. Moreover, to achieve a balanced utilization of the data, the dataset was partitioned into training, validation, and testing subsets in the proportions of 70%, 20%, and 10%, respectively.

In contrast, the MPII human pose dataset—widely recognized as the state-of-the-art benchmark for evaluating articulated human pose estimation in the computer vision field—is dedicated to 2D human keypoint-detection tasks. This dataset comprises approximately 25 K images featuring over 40 K individuals and covering 410 distinct human activities, thereby providing an excellent comparative platform for our experiments. By leveraging the MPII dataset, we were able to compare the performance of various algorithms under complex background conditions, comprehensively assessing the adaptability and robustness of the proposed method. Furthermore, the comparative analysis with the MPII dataset offers an intuitive demonstration of the strengths and weaknesses of our approach in keypoint detection and pose estimation, thereby furnishing valuable insights for subsequent optimization efforts.

In summary, the joint experimentation on both the self-constructed and public datasets not only validates the effectiveness of the proposed method for single-arm robotic manipulator human pose estimation but also provides new data support and technical insights for future research in related fields. Future work will focus on further expanding the dataset’s coverage and exploring additional keypoint annotation strategies, with the aim of enhancing the accuracy and stability of pose estimation in increasingly complex environments.

### 4.2. Experiment Setup

In this experiment, we conducted a systematic study of the human pose-estimation task within a federated learning framework. We allocate 70% of the self-constructed dataset as the training set, 20% as the validation set, and 10% as the test set. The experimental platform consists of one server and ten clients. The dataset is first preprocessed and partitioned on the server and then distributed to each client according to predefined weights. During each round of model aggregation, the system randomly selects five clients, each of which performs five local training epochs.

The model used in this experiment is an improved PoseResNet based on ResNet50, in which deconvolutional layers are added to upsample feature maps and a convolutional layer is appended after the deconvolution modules to generate the final output heatmaps. The network implements standard ResNet blocks (BasicBlock and Bottleneck) while employing a carefully designed deconvolution configuration. The number of keypoints is set to 3, the input image size is 256 × 256, the heatmap size is 64 × 64, the number of deconvolution layers is 3, each with 256 channels and a kernel size of 4.

For our experimental scenario, we designed a specific task to capture the standing poses of individuals wearing a single-arm manipulator, thereby emulating the real-time monitoring of patient movements during robot-assisted rehabilitation. During data preprocessing, we performed a non-IID partition based on predefined client sample-size weights to simulate data quantity heterogeneity across clients, thereby evaluating the robustness and optimization capability of FedGH under heterogeneous data conditions.

As the evaluation metric, we adopt the average PCK as the primary criterion for the human pose-estimation task. The PCK quantifies the localization accuracy of predicted keypoints by measuring the Euclidean distance between predicted and ground-truth keypoints and determining whether each falls within a specified threshold. Given its high recognition precision and credibility in the pose-estimation community, average PCK is used as the main comparison metric in this study.

To investigate the influence of different federated aggregation algorithms on the performance of human pose estimation and keypoint detection, the experiment was designed with the following four groups of comparison and ablation experiments:

1. Train using FedAvg, FedProx, FedDyn, FedBN, and FedAW on the self-constructed dataset;
2. Train using FedAvg, FedProx, FedDyn, FedBN, and FedAW on the MPII dataset;
3. Based on FedAW, apply the improved FedGH strategy to train on the self-constructed dataset;
4. Based on FedAW, apply the improved FedGH strategy to train on the MPII dataset.

Among these, FedAvg, as a widely adopted federated aggregation method, updates the global model by weighted averaging of client model parameters; FedBN addresses data-distribution heterogeneity by updating each client’s batch normalization layers independently, thereby better preserving local feature statistics; FedProx introduces a proximal regularization term in the client’s local objective to effectively balance deviations between local updates and the global model; and FedAW dynamically adjusts aggregation weights based on reliability metrics of client updates and inter-client data-distribution differences, enabling differentiated information fusion; and FedDyn leverages a dynamic regularization-correction term to adaptively align local gradients with the global objective, thereby mitigating update conflicts and improving convergence under non-IID data distributions.

For all methods, the server employs the Adaptive Moment Estimation (Adam) optimizer with a fixed learning rate of 0.001 over 100 communication rounds. During local client updates, either Adam or Stochastic Gradient Descent (SGD) is used as the optimizer—with learning rates varied between $1\times 10^{-4}$ and $1\times 10^{-2}$ according to experimental settings—to assess the impact of different hyperparameters on algorithm performance. The global aggregation batch size is uniformly set to 32.

### 4.3. Results

In this section, we first validate the effectiveness of the proposed FedGH method on the human pose-estimation task and then conduct an in-depth analysis of its improvement in detection accuracy for each keypoint based on the PCK metric.

#### 4.3.1. Effectiveness of FedGH

In the human pose-estimation task, our goal is to train a regression model to predict the precise locations of all human keypoints; to this end, we conduct 100 rounds of federated learning communication between clients and the server to obtain the final global model, and we measure the binary detection performance of each keypoint position using the detection accuracy output by a keypoint heatmap regression model. To compare the impact of different federated learning methods on human pose-estimation performance, we select five commonly used algorithms—FedAvg, FedBN, FedProx, FedDyn, and FedAW—and perform detailed comparative experiments on both our self-constructed dataset and the MPII dataset.

**Figure 3:**
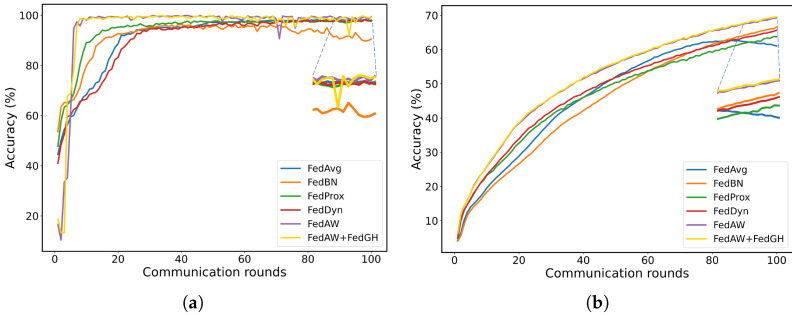
(**a**) The accuracy curves of different federated learning methods under the self-constructed dataset. (**b**) The accuracy curves of different federated learning methods under the MPII dataset.

Figure 3a,b show the accuracy curves of the keypoint heatmap regression model for FedAvg, FedBN, FedProx, FedDyn, FedAW and FedGH on the self-constructed dataset and the MPII dataset, respectively. On the self-constructed dataset, FedGH approaches its maximum accuracy within only 10 rounds and thereafter remains stably within the 98–100% range, achieving twice the convergence speed of traditional methods such as FedAvg, FedBN, and FedProx. In contrast, FedAW consistently underperforms FedGH during the first 10 rounds and exhibits greater accuracy fluctuations thereafter, remaining below FedGH in most rounds. FedAvg, FedBN, and FedProx converge slowly and even show accuracy degradation in later rounds, indicating their inability to tolerate gradient conflicts among heterogeneous client models. By comparison, FedDyn—a state-of-the-art federated learning algorithm recently proposed to address non-IID data—requires roughly 30 rounds to reach comparable accuracy levels and still shows larger oscillations around its plateau. Despite its design to mitigate client drift, FedDyn falls short of FedGH’s rapid stabilization and peak performance. These results demonstrate that FedGH, through its gradient-coordination mechanism, effectively mitigates gradient conflicts among heterogeneous clients, thereby achieving faster and more stable convergence of the global model.

To quantify the computational and communication overhead introduced by FedGH’s gradient projection and parameter reconstruction, we measured end-to-end training durations under identical hardware and network conditions on the self-constructed dataset for 100 communication rounds. FedAvg required a total of 83,490.4 s, whereas FedGH completed training in 75,893.1 s—a reduction of approximately 9.1%. Although FedGH executes PCGrad projection and parameter reconstruction in each round, these operations incur only a modest per-round cost relative to a full forward/backward pass. Moreover, FedGH stabilizes within 10 rounds versus over 50 rounds for FedAvg, more than compensating for any extra computation. Communication overhead remains essentially unchanged, since both methods exchange gradients of identical dimensionality at each synchronization step. Therefore, FedGH not only enhances detection performance under non-IID settings but also delivers comparable—or even reduced—overall training time, underscoring its practical scalability in privacy-sensitive, distributed deployments.

To further validate FedGH’s applicability in a more general and complex scenario, we applied the same experimental settings to the MPII dataset. As shown in Figure 3b, FedGH achieved a 1–2 percentage-point accuracy gain over FedAW after approximately 20 rounds and continued to outperform the other methods by 3–6 percentage points throughout the remaining training, reaching a peak accuracy of nearly 69%. In comparison, FedBN only began to catch up after about 80 rounds but still exhibited slower overall convergence; FedProx’s strong constraints on local updates led to a plateau in accuracy improvements in later rounds; and FedAvg experienced a marked accuracy decline to 61% in the final stages. FedDyn’s accuracy curve remained consistently below that of FedGH, with the performance gap widening as training progressed. Although FedDyn initially outperformed FedBN, it was overtaken by FedBN at around round 80. Ultimately, neither FedDyn nor FedAW could match FedGH’s stabilization speed or its superior final accuracy. These results indicate that even under the highly heterogeneous data distributions and pronounced sample diversity of the MPII scenario, FedGH’s gradient harmonization strategy effectively promotes cooperative updates among client models, substantially enhancing both convergence efficiency and final performance of the global model.

**Figure 4:**
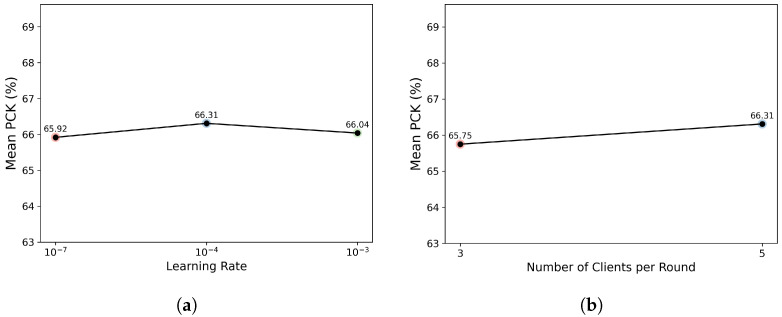
(**a**) Effect of varying the global learning rate ($10^{-7}$, $10^{-4}$, $10^{-3}$) on the final mean PCK of FedGH. (**b**) Effect of varying the number of clients participating per round (3 vs. 5) on the final mean PCK of FedGH.

To evaluate the robustness of FedGH to hyperparameter configurations, we conducted two sensitivity analyses. As illustrated in Figure 4a, on the MPII dataset, we varied the global learning rate among $10^{-7}$, $10^{-4}$, and $10^{-3}$ while fixing the number of participating clients at 5 per round. The resulting mean PCK values remained within a narrow range (65.92–66.31%), suggesting that FedGH exhibits strong stability to fluctuations in learning rate. In Figure 4b, on the MPII dataset, we fixed the learning rate at $10^{-4}$ and compared the effects of selecting 3 versus 5 clients per communication round. The mean PCK was 65.75% when 3 clients participated per round, and 66.31% when 5 clients participated, resulting in a difference of only 0.56 percentage points. Overall, these results demonstrate that FedGH consistently achieves stable accuracy under varied training conditions, underscoring the robustness and practical applicability of its gradient harmonization strategy across a wide spectrum of hyperparameter settings.

#### 4.3.2. Human Pose Estimation

To quantitatively evaluate the localization accuracy of different methods, we adopt the as the evaluation metric, which uses a normalized Euclidean distance threshold to judge continuous localization precision. computes the Euclidean distance between each predicted keypoint and its corresponding ground truth, divided by a normalization factor. To ensure comparability of experimental metrics, we employ an adaptive threshold strategy on both datasets, dynamically adjusting the normalization factor based on image diagonal length and upper-arm or forearm length: specifically, we use the image diagonal length for the shoulder and the corresponding upper-arm and forearm lengths for the elbow and wrist, respectively, and apply adaptive thresholds of 0.3, 0.4, and 0.5 times the normalized distance to determine correct localization. This adaptive strategy accommodates scale differences among joints and provides a more representative evaluation of keypoint accuracy.

**Figure 5:**
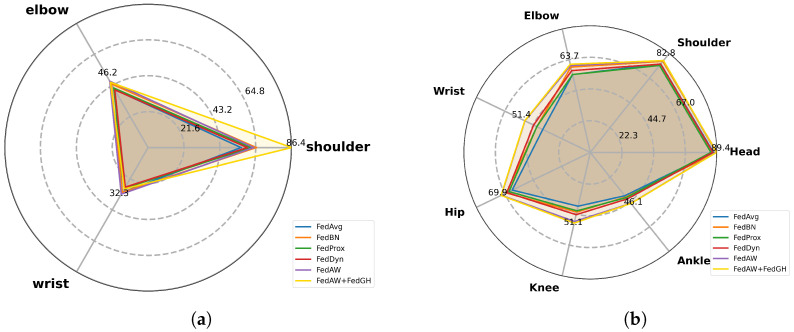
(**a**) The spider chart of keypoint PCK values for FedGH and federated learning methods, comparing human pose-estimation performance on the self-constructed dataset. (**b**) The spider chart of keypoint PCK values for FedGH and federated learning methods, comparing human pose-estimation performance on the MPII dataset.

Figure 5a,b present the spider plots of the maximum PCK values for six federated learning methods on representative keypoints, evaluated on our self-constructed dataset and the MPII dataset, respectively. On the self-constructed dataset, FedGH achieved PCKs of 86.4%, 45.4%, and 28.7% at the shoulder, elbow, and wrist, representing an average improvement of approximately 2–3 percentage points over FedAW and 4–6 percentage points over FedAvg, FedBN, and FedProx. Notably, FedGH’s shoulder exceeded that of FedBN by 21.2% and that of FedAvg by 30.4%. Compared to FedDyn—widely recognize as the state-of-the-art federated learning method for non-IID scenarios—FedGH nevertheless outperforms it by 26.6% at the shoulder, 5.3% at the elbow, and 1.3% at the wrist. This advantage primarily stems from the gradient-coordination mechanism: during aggregation, FedGH automatically aligns gradient directions across clients, mitigating update conflicts arising from data heterogeneity and yielding more consistent responses to joint boundaries and complex pose features, thereby achieving higher detection precision.

On the more challenging MPII dataset, FedGH likewise delivered comprehensive performance gains. It attained shoulder, elbow, and wrist PCKs of 82.9%, 63.7%, and 51.4%, leading FedAW by approximately 1–2 percentage points and outperforming FedBN and FedProx by 3–5 percentage points. Specifically, FedGH achieved an ankle PCK of 46.06%, surpassing FedAW by 0.12 percentage points and outperforming FedBN, FedProx, and FedAvg by 5.32–6.24 percentage points. Moreover, FedGH’s wrist PCK reached 51.38%, edging out FedAW by 0.14 percentage points and exceeding FedBN, FedProx, and FedAvg by 9.47–14.10 percentage points. When compared to FedDyn—one of the most advanced federated learning algorithms designed for non-IID scenarios—FedGH outperforms it by 3.2% at the shoulder, 4.6% at the elbow, and 6.9% at the wrist, while also leading by 2.6% on the head and 4.1% on the ankle. Although the MPII dataset’s complex backgrounds and diverse poses can weaken the effectiveness of local updates, FedGH alleviates the disturbance of noisy samples by sharing gradient information across clients and guiding all parties toward a superior global direction, thus enhancing the stability of joint position predictions.

In Table 1, we compare the Head-normalized Percentage of Correct Keypoints at threshold 0.5 (PCKh@0.5) performance of FedGH on the MPII dataset with three classical centralized training methods [29,30,31]. In terms of average PCK, FedGH achieves 66.3%, which is substantially higher than the early hand-crafted-feature-based approaches of Pischulin et al. [29] yet still lags behind the end-to-end convolutional regression framework of Tompson et al. [30]. On individual keypoints, FedGH attains 82.8% and 63.7% PCK at the shoulder and elbow, respectively—improvements of 33.8 and 22.9 percentage points over Pischulin et al. [29]—demonstrating strong localization capability for mid-scale joints; at the wrist, it reaches 51.4%, similarly outperforming both centralized feature methods by roughly 17 percentage points. Moreover, the recent transformer-based approach of Ren et al. [31] establishes a new centralized baseline for 2D human pose estimation, achieving an average PCK as high as 87.9% on the MPII dataset. It reports 94.9% at the shoulder, 88.3% at the elbow and 81.8% at the wrist. This highlights the advantage of a centralized training regime, where access to the full MPII annotations and global data distribution enables the model to learn richer representations and achieve markedly higher PCK scores.

By contrast, Tompson et al. [30] maintain superiority on most keypoints (e.g., head at 95.8%, shoulder at 90.3%) and exceed FedGH on smaller joints (e.g., ankle at 62.8%), while the transformer-based baseline of Ren et al. [31] also outperforms FedGH across all major joints. This reflects the inherent advantage of centralized models in leveraging large-scale annotations and global feature learning. Indeed, centralized training represents the upper bound on accuracy when data can be fully aggregated. Federated learning, by design, forgoes this centralized advantage in order to preserve client privacy and reduce communication of raw data. Consequently, FedGH’s performance should be understood as a trade-off: it attains competitive accuracy under strict non-IID and privacy constraints but does not—and by principled constraints cannot—surpass the centralized upper bound. Overall, FedGH offers a viable compromise, delivering strong joint-detection accuracy while guaranteeing distributional privacy and communication efficiency in environments where data centralization is infeasible.

**Figure 6:**
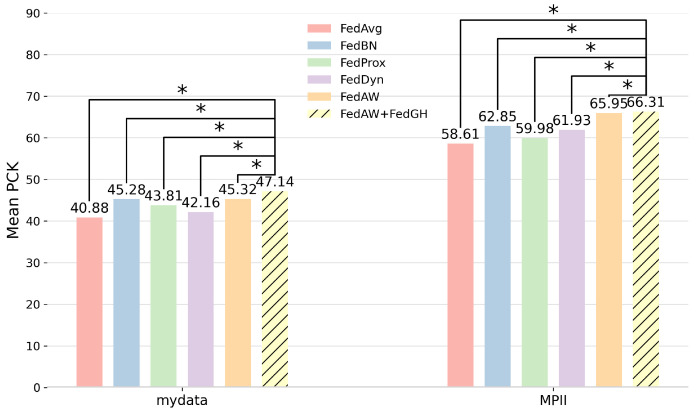
Comparison of average PCK values for human pose estimation between FedGH and various federated learning methods on self-constructed and MPII datasets. An asterisk (∗) denotes that the paired *t*-test achieves *p* < 0.05.

Finally, Figure 6 summarizes the maximum mean PCK values achieved by the six federated learning methods on both the self-constructed dataset and the MPII dataset. On the self-constructed dataset, FedGH attained a maximum mean PCK of 47.14, representing a 1.82 percentage points improvement over FedAW. On the more challenging MPII dataset, FedGH likewise achieved a mean PCK of 66.31, surpassing FedAW by 0.36 percentage points and outperforming traditional methods by 3–7 percentage points. These results demonstrate that, whether in the relatively homogeneous self-constructed scenario or in the complex, diverse MPII setting, FedGH consistently enhances overall model performance through its cross-client gradient-coordination mechanism. It accelerates early convergence and consolidates the optimal solution in later rounds, fully validating the strong adaptability and superiority of FedGH in handling non-IID data. Connector lines link the FedGH bar to each corresponding baseline, and an asterisk (∗) denotes that the paired t-test achieves p<0.05. For example, on the self-constructed dataset, the FedGH–FedProx comparison is marked with ∗ (p=0.01), and all other FedGH–baseline pairs on both datasets likewise satisfy the p<0.05 threshold.

To evaluate the statistical significance of FedGH on average PCK, we performed paired t-tests between FedGH and FedAvg, FedBN, FedProx, FedDyn, and FedAW using the 100 rounds of average PCK results on both our self-constructed dataset and MPII dataset, as shown in Table 2 and Table 3. A larger t-value implies a smaller *p*-value and higher significance. On the self-constructed dataset, the *p*-values for FedGH versus FedAvg, FedBN, FedProx, FedDyn, and FedAW are $2.7565\times 10^{-3}$, $3.8564\times 10^{-10}$, $9.8940\times 10^{-3}$, $7.6021\times 10^{-3}$, and $5.2057\times 10^{-5}$, all below the significance level α=0.05, indicating a statistically significant PCK improvement by FedGH. On the more challenging MPII dataset, all *p*-values are smaller than $1\times 10^{-22}$—specifically $3.2879\times 10^{-45}$, $6.1387\times 10^{-44}$, $2.5023\times 10^{-60}$, $2.0794\times 10^{-64}$, and $5.5658\times 10^{-23}$—further confirming FedGH’s highly significant advantage under complex, diverse human-pose conditions. These tests strongly support that FedGH’s gradient-harmonization mechanism accelerates convergence and improves overall performance in non-IID settings.

Although our self-constructed single-arm robotic dataset offers high-quality collection and annotation, its sample size is relatively limited and its range of actions and viewing angles is narrow, which may not fully reflect the diversity of human poses in real scenarios. Consequently, some keypoints achieve high PCK and accuracy on the self-constructed dataset, but the average PCK across all keypoints remains lower than that on MPII. Further validation with larger-scale, multi-angle, and multi-subject data is therefore required to assess the generalization ability of FedGH under complex backgrounds and large-scale human populations.

## 5. Conclusions

This work addresses the problem of gradient conflicts in federated learning for non-independent and identically distributed human pose-estimation scenarios by proposing a gradient-coordination-based aggregation strategy, FedGH. Experimental results demonstrate that FedGH achieves faster convergence and higher keypoint-detection accuracy on both a self-constructed single-arm robotic-assisted rehabilitation dataset and the MPII public dataset, with average PCK improving to 47.14% and 66.31%, respectively—gains of 1.82 and 0.36 percentage points over the existing FedAW method.

While these results preliminarily validate the effectiveness of gradient coordination in moderately non-IID scenarios, full robustness claims require further testing under more extreme non-IID conditions, such as client-specific label bias and inter-client feature drift. Moreover, although FedGH’s PCK is below the centralized state of the art (79.6% by Tompson et al. [30] in Table 1), it offers a privacy-preserving, communication-efficient solution suitable for distributed deployments where raw data sharing is not permissible.

Building on prior hypotheses regarding gradient conflict, FedGH leverages PCGrad projection and parameter-reconstruction mechanisms to effectively mitigate the mutual cancellation of client gradients, thereby validating the importance of gradient coordination in non-IID settings. These findings not only enrich the theoretical framework of federated aggregation strategies but also offer a practical solution for distributed pose estimation in applications such as medical rehabilitation and action analysis.

However, the self-constructed dataset is inherently limited by its small scale (only 2060 images of a single subject) and lack of pose diversity (restricted to single-arm configurations and upright stances). Such constraints may impede the model’s ability to generalize to varied human motions and multi-subject scenarios, potentially resulting in lower PCK performance for under-represented keypoints in more complex environments. To address these shortcomings, future work will incorporate larger-scale, multi-subject, multi-view datasets such as COCO-WholeBody and CrowdPose, which present a wider range of keypoints, complex articulations, occlusions and multi-view settings. This will allow us to rigorously assess FedGH’s generalization and robustness under truly heterogeneous, non-IID distributions, and further refine our gradient-coordination mechanisms for full-body, distributed pose estimation.

Future work may explore multi-task joint learning, dynamic projection correction, and personalized adaptive weight allocation to tackle higher-dimensional keypoint estimation and more complex network architectures under heterogeneous data distributions.

## Figures and Tables

**Table 1:** Comparison of PCK performance of FedGH and other centralized training baselines.

Method	Avg	Hea	Sho	Elb	Wri	Hip	Kne	Ank
Pischulin et al. [29]	44.1	74.3	49.0	40.8	34.1	36.5	34.4	35.2
Tompson et al. [30]	79.6	95.8	90.3	80.5	74.3	77.6	69.7	62.8
Ren et al. [31]	87.9	96.4	94.9	88.3	81.8	88.2	83.0	78.3
FedGH	66.3	89.1	82.8	63.7	51.4	69.9	51.1	46.1

**Table 2:** Paired-sample t-test results comparing FedGH with other federated learning methods on the self-constructed dataset, indicating the significance of differences in average PCK.

Method	t-Value	*p*-Value
FedGH vs. FedAvg	3.07	0.003
FedGH vs. FedBN	6.95	<0.001
FedGH vs. FedProx	2.63	0.010
FedGH vs. FedDyn	2.73	<0.001
FedGH vs. FedAW	4.23	<0.001

**Table 3:** Paired-sample t-test results comparing FedGH with other federated learning methods on the MPII dataset, indicating the significance of differences in average PCK.

Method	t-Value	*p*-Value
FedGH vs. FedAvg	25.44	<0.001
FedGH vs. FedBN	24.58	<0.001
FedGH vs. FedProx	37.51	<0.001
FedGH vs. FedDyn	41.49	<0.001
FedGH vs. FedAW	12.93	<0.001

## Data Availability

The data presented in this study are available on request from the corresponding author. The data are not publicly available due to privacy and ethical restrictions.
